# *Dpe2/phs1* revealed unique starch metabolism with three distinct phases characterized by different starch granule numbers per chloroplast, allowing insights into the control mechanism of granule number regulation by gene co-regulation and metabolic profiling

**DOI:** 10.3389/fpls.2022.1039534

**Published:** 2022-11-01

**Authors:** Xiaoping Li, Ardha Apriyanto, Junio Flores Castellanos, Julia Compart, Sidratul Nur Muntaha, Joerg Fettke

**Affiliations:** Biopolymer Analytics, Institute of Biochemistry and Biology, University of Potsdam, Potsdam-Golm, Germany

**Keywords:** LCSM, RNA-Seq, metabolic-profiling, starch granule number regulation, starch initiation, starch degradation

## Abstract

An Arabidopsis mutant lacking both the cytosolic Disproportionating enzyme 2 (DPE2) and the plastidial glucan Phosphorylase 1 (PHS1) revealed a unique starch metabolism. *Dpe2/phs1* has been reported to have only one starch granule number per chloroplast when grown under diurnal rhythm. For this study, we analyzed *dpe2/phs1* in details following the mutant development, and found that it showed three distinct periods of granule numbers per chloroplast, while there was no obvious change observed in Col-0. In young plants, the starch granule number was similar to that in Col-0 at first, and then decreased significantly, down to one or no granule per chloroplast, followed by an increase in the granule number. Thus, in *dpe2/phs1*, control over the starch granule number is impaired, but it is not defective in starch granule initiation. The data also indicate that the granule number is not fixed, and is regulated throughout plant growth. Furthermore, the chloroplasts revealed alterations during these three periods, with a partially strong aberrant morphology in the middle phase. Interestingly, the unique metabolism was perpetuated when starch degradation was further impaired through an additional lack of Isoamylase 3 (ISA3) or Starch excess 4 (SEX4). Transcriptomic studies and metabolic profiling revealed the co-regulation of starch metabolism-related genes and a clear metabolic separation between the periods. Most senescence-induced genes were found to be up-regulated more than twice in the starch-less mature leaves. Thus, *dpe2/phs1* is a unique plant material source, with which we may study starch granule number regulation to obtain a more detailed understanding.

## Introduction

In plant chloroplasts, transitory starch is the essential carbohydrate, which is assimilated *via* photosynthesis during the day. Each chloroplast in *Arabidopsis thaliana* (L.) Heynh. wild-type leaves contains three to seven discoid starch granules, and the starch stored during the day is nearly completely depleted by dawn (Streb and Zeeman, 2012; Sulpice et al., 2014). The precise degradation rate of starch seems to be critical for normal plant growth, ensuring that there is substantial energy to fuel fundamental metabolic processes and plant development during the night (MacNeill et al., 2017). Although the regulation mechanism underlying the degradation rate is unknown, evidence exists that both the starch granule number per chloroplast and granule morphology are important parameters. It has been shown that mutants with a decreased starch granule number per chloroplast (*e.g.*, *ss4*, *ptst2*, and *pii1*) have a lower starch degradation rate (Crumpton-Taylor et al., 2013; Seung et al., 2017; Vandromme et al., 2019). Further, in Arabidopsis, the starch granule morphology and number are independently regulated (Crumpton-Taylor et al., 2012; Malinova et al., 2018); however, the molecular basis for the regulation of the normal starch granule number per chloroplast remains obscure. Research has largely focused on identifying the role of a single related protein or enzyme involved in starch initiation or degradation in the past. However, there are various open questions, thus *e.g*., how the plant maintains the normal starch granule numbers; is the number controlled throughout the plant growth, and to which extent starch degradation is also involved?

More than 40 proteins have been reported to be involved in starch metabolism (Streb and Zeeman, 2012; Stitt and Zeeman, 2012). Starch synthase 4 (SS4; [EC: 2.4.1.21]) is the widely-accepted initiation enzyme, which transfers the glucosyl residue from the donor ADP-glucose to a not yet defined short oligosaccharide primer (Roldan et al., 2007; Szydlowski et al., 2009; Crumpton-Taylor et al., 2013). Further potential proteins interacting with SS4 include Protein targeting to starch 2 (PTST2, At1g27070), which is thought to donate oligosaccharides for SS4; MAR-binding filament-like protein 1 (MFP1, At3g16000); Myosin-resembling chloroplast protein (PII1, At4g32190); and Starch synthase 5 (SS5, AT5G65685) (Seung et al., 2017; Seung et al., 2018; Vandromme et al., 2019; Abt et al., 2020). All respective single mutants had a reduced starch granule number per chloroplast. PHS1, the plastidial phosphorylase ([EC: 2.4.1.1]), adds or releases glucosyl residues from the non-reducing end of glucan chains, in principle suggesting it plays roles in both synthesis and degradation (Fettke et al., 2012; Hwang et al., 2020). Evidence that PHS1 is involved in the regulation/initiation of starch granules also exists (Satoh et al., 2008; Nakamura et al., 2012); for example, the plastidial phosphorylase is involved in starch initiation in the rice endosperm (Satoh et al., 2008; Hwang et al., 2010; Hwang et al., 2016). However, a single mutant *phs1* in Arabidopsis has presented normal starch granule numbers (Malinova et al., 2014).

A very interesting mutant is *dpe2/phs1* lacking both PHS1 and DPE2, the cytosolic disproportionating enzyme 2 (EC: 2.4.1.25), for which an uneven starch distribution has been revealed, with nearly starch-free mature leaves and young leaves having mostly one large and spherical starch granule per chloroplast when grown under circadian periodicity (Malinova et al., 2014; Malinova and Fettke, 2017). Under continuous light, *dpe2/phs1* revealed a similar number of starch granules per chloroplast, compared to wild-type (Malinova et al., 2014). A triple mutant, *dpe2/phs1/ss4*, had only one starch granule per chloroplast, which was huge and almost perfectly spherical (Malinova et al., 2017), showing that the existing starch granules are formed independently of both PHS1 and the SS4 pathway. Furthermore, it has been shown that a total block in starch degradation (*via* an additional lack of α-Glucan, water dikinase; GWD [EC 2.7.9.4]) in *dpe2/phs1/sex1-8* (Malinova and Fettke, 2017) restored the starch granule number to that in the wild-type; whereas, in case of reduced starch degradation, the starch granule number was still reduced (Muntaha et al., 2022).

Similar to the mature leaves of *dpe2/phs1*, several mutants impaired in starch metabolism have also revealed affected chloroplast stability. The single mutant *mex1*, lacking the maltose exporter (AT5G17520), accumulated a high amount of maltose inside the chloroplasts and showed severe chloroplast degradation in mature leaves (Stettler et al., 2009). It has also been reported that the starchless mutants *pgm1* (Phosphoglucomutase 1 [EC 5.4.2.2]) and *adg1* (ADP-glucose pyrophosphorylase 1 [EC 2.7.7.27]) revealed higher Rubisco-containing bodies, leading to higher percentages of aberrant chloroplasts (Izumi et al., 2010; Zhuang and Jiang, 2019). This points to a link between chloroplast performance and the central starch metabolism, although the nature of this link is unknown.

In this study, we found the double mutant *dpe2/phs1*, loses the control over the starch granule number per chloroplast during developing and the starch granule number alters in three distinct periods. Both RNA-seq analysis and GC-MS metabolic profiling of these periods of *dpe2/phs1* provide insights to further our understanding of starch granule number regulation.

## Materials and methods

### Plant materials and growth conditions

*Arabidopsis thaliana* seeds were sterilized using 6% [v/v] hypochlorous acid containing 0.02% [v/v] Tween-20 and sown on Murashige and Skoog (MS) medium with 0.8% [w/v] agar and 1% [w/v] sucrose, pH 5.7. The plates were vernalized in darkness at 4°C for three days. Seedlings were transplanted to soil after 7–10 days and grown in a light/dark regime (12 h light, 20°C, 110 μmol m^-2^s^-1^; 12 h dark, 16°C; 60% relative humidity).

The crossing of mutant *dpe2/phs1* was addressed previously (Malinova et al., 2014). To determine the fresh weight and leaf number, more than nine mutant plants were used per week till flowering.

*Sex4-3* (SALK_102567) (Niittyla et al., 2006), and *isa3* (GABI_280G10) (Delatte et al., 2006) in the Col-0 background have been described previously. The knockout lines *dpe2/phs1/sex4* and *dpe2/phs1/isa3* were generated by crossing the *dpe2/phs1* mutant with the single mutants *sex4* and *isa3*, respectively, and self-pollinating the F1 generation.

The activities of phosphorylases and DPE2 were detected by native gels and zymograms, as described previously (Fettke et al., 2005; Malinova et al., 2014). Analysis of the SEX4 protein was performed by Western blot, as previously described (Niittyla et al., 2006). T-DNA insertion of ISA3 was confirmed by PCR (Forward: 5’-ACATCAAATCTTTACGTACTCGGC-3’ and Reverse: 5’-ACCTTATTTTTCCTCTACCGTGCG-3’; T-DNA specific primer LB o8474: 5’-ATAATAACGCTGCGGACATCTACATTTT-3’).

### Laser confocal scanning microscopy (LCSM)

LCSM imaging was performed as described by Liu et al. (2021). In brief, leaves from different ages of plant were harvested and immersed in safranin O solution (5g/L) for 20 min. The starch and chlorophyll signals were visualized with a Zeiss LSM 880 Airyscan confocal microscope.

### *In situ* staining of starch

Shoots were stained as described by Malinova et al. (2014). The harvested shoots were decolored in 80% (v/v) ethanol at 80°C, afterwards the pale shoots were stained with iodine solution. The starches in the root tips were observed by staining in iodine solution for twenty seconds.

### Starch quantification and morphology analysis by scanning electron microscopy (SEM)

Starch was enzymatically quantified according to Malinova et al. (2014). Fifty milligram of fresh leaf materials were used. The homogenized leave material was washed with 80% (v/v) ethanol and the pelleted starch was solubilized and hydrolyzed by amyloglucosidase. The resulting glucose was measured enzymatically. Native starch granules were isolated and SEM analysis was performed as described by Malinova et al. (2017).

### Chain-length distribution analysis of starch and maltodextrin

Starch granules (10 mg) were used and digested as described by Mahlow et al. (2014). In brief, 10 mg of starch was solubilized by heating at 99°C for 10 min and 2 U isoamylase was used to hydrolyze the soluble starch at 40°C.

For quantitation of the maltodextrin, 100 mg of leaves were frozen in liquid nitrogen and homogenized with a mortar. Then, 500 µL cold 20% [v/v] ethanol was added to each sample and mixed by vigorously shaking. After centrifugation at 4°C, 14,000 rpm for 10 min, the supernatant was collected and heated to 99°C for 10 min. Followed by centrifugation, the supernatant containing the maltodextrin was recovered and concentrated by drying in a speed vacuum centrifuge (Thermo Savant SPD111V-115).

The starch chain length distribution and the maltodextrins were analyzed by capillary electrophoresis equipped with laser-induced fluorescence detection (CE-LIF), as described by Malinova et al. (2014). Following determination of the reducing ends, 100 nmol glucans were used for APTS (8-Aminopyrene-1,3,6-Trisulfonic Acid, Trisodium Salt) labeling. Note that the glucose and maltose content was excluded for the maltodextrin content determination by calculation according to capillary electrophoresis result.

### RNA-seq analysis

The leaves of *dpe2/phs1* and Col-0 were harvested after 9 h illumination for each period. Total RNAs were extracted using TRIzol reagent (Invitrogen, US), according to the manufacturer’s instructions. A NanoPhotometer^®^ spectrophotometer was used to assess the purity of the RNA (IMPLEN, CA, USA). The Bioanalyzer 2100 system’s RNA Nano 6000 Assay Kit was used to examine RNA integrity and for quantification (Agilent Technologies, CA, USA). Library construction and sequencing with an Illumina novaseq 6000 sequencer were performed by Novogene Corporation (UK). Annotation files for the reference genome and gene models were downloaded directly from the genome website browser (NCBI/UCSC/Ensembl). The HISAT2 software was used to map paired-end clean reads to the reference genome. The collected RNA-seq data were deposited in the NCBI GEO repository, under the accession number GSE201804. The DESeq2 R software was used to perform differential expression analysis between two conditions/groups (three biological replicates per condition). The False Discovery Rate (FDR) was controlled by adjusting *P*-values using Benjamini and Hochberg’s method. Genes discovered by DESeq2 with an adjusted *P*-value < 0.05 were labeled as differentially expressed. The statistical enrichment of differentially expressed genes in KEGG pathways was tested using the clusterProfiler R program.

### qRT-PCR analysis

The RNAs isolated with TRIzol reagent (Invitrogen, US) were reverse transcribed into cDNA using a first cDNA synthesis kit (ThermoFisher, US), following the manufacturer’s instructions. qRT-PCR was performed with SYBR Green PCR Master Mix (Applied Biosystems), following the manufacturer’s instructions. Reactions were carried out in triplicate. Relative expression levels were determined by the comparative threshold cycle (ΔΔCt) method, using ACTIN as a reference gene. The used primers are given in **Table S4**.

### Metabolite profiling

The same leaf materials from the three periods (50 mg each) used for RNA-seq were used for metabolite profiling, using Gas Chromatography-Mass Spectrometry (GC-MS). The following data analysis was conducted according to Muntaha et al. (2022).

## Results

### *Dpe2/phs1* lost the control over starch granule number during growth and the starch granule number per chloroplast fluctuates in three distinguishable periods

**Figure 1:**
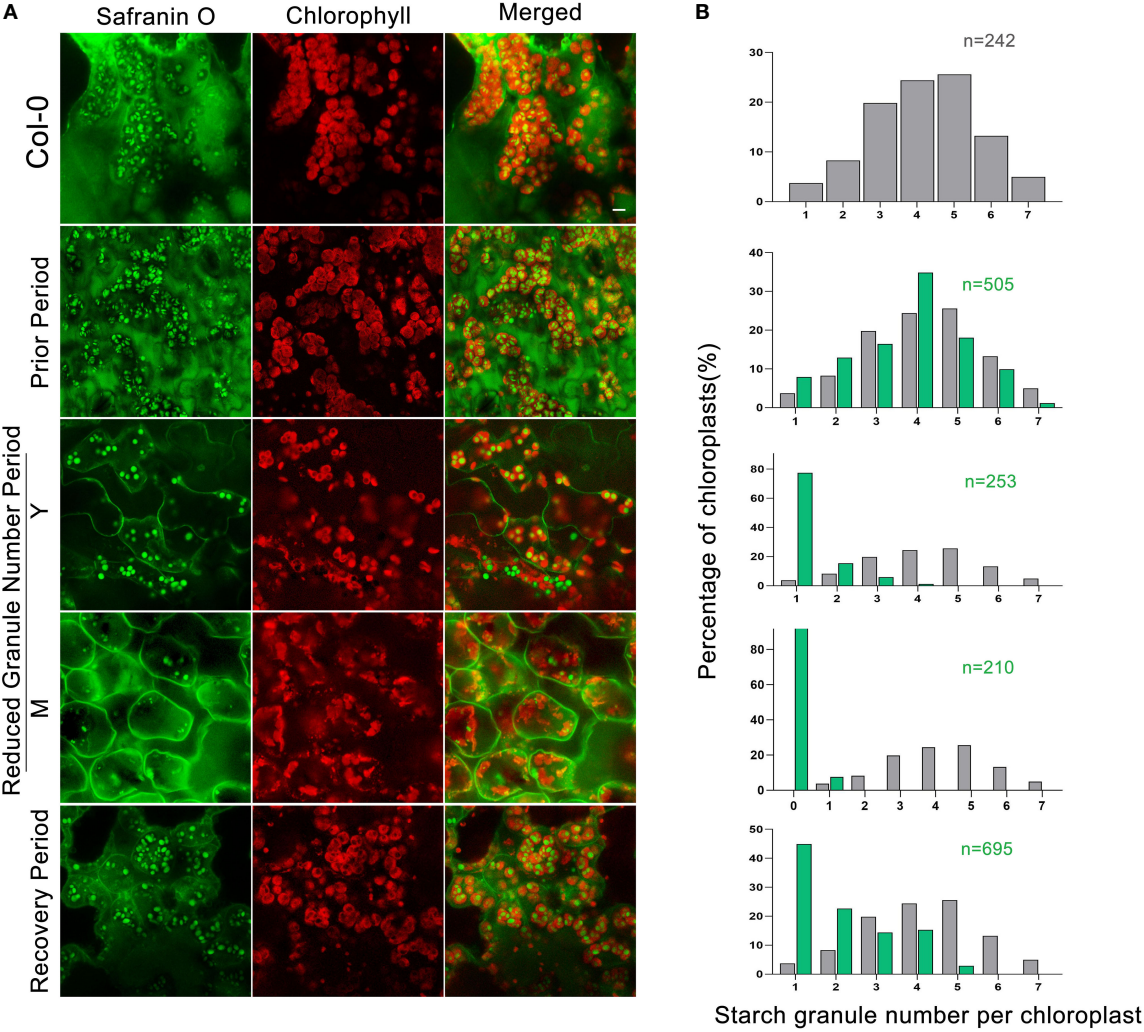
LCSM analysis of starch granules in chloroplasts in three periods: **(A)** Leaves of Col-0 taken from the fifth week. Prior Period leaves were taken from three-week-old rosettes of *dpe2/phs1*. Young (Y) and mature (M) leaves in the Reduced Granule Number Period (RGN) were taken from five-week-old rosettes of the mutant. Recovery Period leaves were taken at the beginning of the sixth week from the mutant. All samples were taken in the middle of the day (6h of light). Scale bar is 5 µm; **(B)** Quantification of starch granules per chloroplast. Grey and green colors represent Col-0 and *dpe2/phs1*, respectively. N indicates the observed number of chloroplasts. Note: only the granule number distribution of (M) starts with zero.

The double mutant *dpe2/phs1* has been described as having a single starch granule per chloroplast in immature leaves, with virtually no starch in mature leaves when grown under a normal light/dark cycle (Malinova et al., 2014; Malinova and Fettke, 2017). We used a modified LCSM (laser confocal scanning microscopy) technique combined with safranin O staining (Liu et al., 2021), allowing for rapid analysis of many samples, in order to determine the starch granule number per chloroplast during plant development. We observed that the starch granule number in *dpe2/phs1* changed during plant development, while no evident changes in Col-0, *phs1* and *dpe2* were detected (**Figures 1A**, **S1A**). Strikingly, *dpe2/phs1* revealed three distinct periods: In the first period, designated as the Prior Period P, similar to Col-0, most chloroplasts had three to five granules, but chloroplasts containing one or two starch granules were more dominant than in Col-0 (**Figure 1B**). For the second period, designated as the Reduced Granule Number Period RGN, the analysis was further separated between mature M and young Y leaves, the latter were the youngest two or three leaves. Approximately 80% of the chloroplasts of immature leaves had only one starch granule (**Figure 1B**), and no chloroplast with more than four granules was detected out of 253. In mature leaves, starch granules were rare and most chloroplasts revealed a highly aberrant morphology, likely indicating degradation, as described previously (Malinova et al., 2014; Malinova and Fettke, 2017; Malinova et al., 2017). Less than 10% of all analyzed chloroplasts contained one large spherical granule. In the last period, the Recovery Period R, the granule number again increased, with most chloroplasts showed one to four starch granules, with 45% of chloroplasts having a single granule, suggesting a regulation of the granule number throughout the plant growth. Moreover, in R, the starch granule size was inhomogeneous, with one large spherical granule being frequently found in chloroplasts. Further, the chloroplast morphology was similar to that in the wild-type and no indication of degradation was observed. Interestingly, the occurrence of RGN varied, among five independent growth batches, this period appeared twice at the end of the third week and three times at the beginning of the fifth week, as confirmed *via* LCSM.

In addition, we followed the starch granule decrease from P to the RGN in detail, and found an inconsistency in starch granule number per chloroplast within the same leaf: the chloroplasts of palisade mesophyll cells decreased to only one starch granule earlier than those in spongy mesophyll cells (**Figure S1B**).

### In RGN, *dp.e2/phs1* revealed heterogeneous starch distribution and impaired growth both in shoot and root

**Figure 2:**
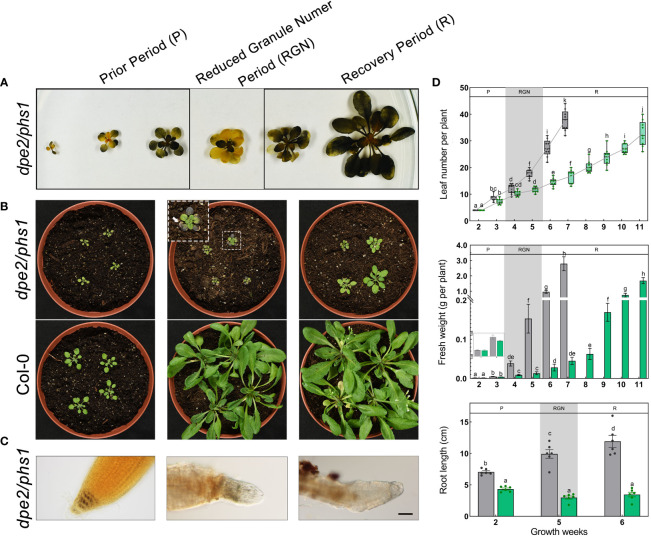
*dpe2/phs1* showed three distinguishable periods when grown under light/dark regime: **(A)** Semi-quantitative starch determination. Shoots harvested at the EOD from two to seven weeks, decolorized shoots were stained with iodine solution; **(B)** Growth phenotype of *dpe2/phs1* from the three periods. Shoots were harvested at three, five, and six weeks, respectively; **(C)** Starch in root caps. The starch in the root caps was stained with I_2_ solution. Bars = 50 μm; **(D)** Growth parameters of *dpe2/phs1*. Plants were analyzed from two weeks until flowering. Grey and green colors indicate Col-0 and *dpe2/phs1*, respectively. Letters indicate statistically significant differences (p ≤ 0.05) among the double mutant and Col-0, according to the one-way ANOVA with Duncan’s *post-hoc* test. More than nine plants were analyzed for determination of leaf number and fresh weight per week. Roots of plants at two, five, and six weeks were used, and six plants each were analyzed. P-Prior Period; RGN-Reduced Granule Number Period; R-Recovery Period.

The observed alterations in the starch granule number per chloroplast were accompanied by changes in leaf starch distribution (**Figure 2A**). During growth, in P, *dpe2/phs1* contained starch in the whole shoot. However, in RGN, the starch was distributed heterogeneously, as described previously (Malinova et al., 2014), and nearly no starch was detected in mature leaves in contrast to young leaves. In R, starch was detected again in the entire shoot. In addition, blight margins were found in mature leaves in RGN (**Figure 2B**). A similar, but less severe, phenotype has been characterized in the parent line *phs1* (Zeeman et al., 2004). However, there was no starch distribution alteration observed in Col-0 during plant growth (**Figure S1C**).

In addition to the reduced starch granule number per chloroplast and the uneven starch distribution, *dpe2/phs1* revealed an obvious decreased development in RGN. The young plants of *dpe2/phs1* had the similar leaf numbers and fresh weights as Col-0, but a significant smaller leaf area, whereas from the fourth to fifth week, compared to Col-0, the mutant showed significant lower fresh weight and a fewer leaf number; moreover, a delayed flowering after eleven weeks of germination was detected (**Figures 2B, D**). Although the occurrence of RGN varies, it was always coupled with a reduction in growth and uneven starch distribution between young and mature leaves. Therefore, for all mutant materials used during this study the period was confirmed prior by LCSM and iodine staining, when more than 80% of tested mutants showed both uneven starch distribution and reduced starch granule numbers, the leaf materials were harvested.

In *dpe2/phs1*, the starch in the roots was also impaired both in RGN and R (**Figure 2C**). The root length of *dpe2/phs1* was shorter than that of Col-0, independent of the period (**Figure 2D**, bottom). However, P showed a normal starch distribution in four layers of the columella cells, whereas deformed and starch-free root caps were detected both in RGN and R (**Figure 2C**).

### Starch content and granule morphology differs in the three distinguishable periods

In Col-0, no significant difference in starch content per fresh weight during growth was observed; thus, Col-0 synthesized around 37 μmol of glucose equivalent starch per fresh weight (g) by the end of the day (EOD), and consumed almost all of it by the end of the night (EON) (**Table 1**). However, *dpe2/phs1* had impaired starch remobilization in all three periods (**Table 1**). In particular, young leaves had twice as much starch as Col-0 at EOD in RGN, but only around 28% of the starch was utilized during the night. In mature leaves, there was still a certain amount of starch and a partial degradation during the night was detected. However, even before RGN—namely, in P—the starch degradation in the night was clearly affected, pointing to increasing effects on starch metabolism during the growth of the plant.

**Table 1:** Starch content (μmol glc. equivalent/g FW) of Col-0 and *dpe2/phs1* grown under a light-dark regime (12h/12h).

Genotype	Period	End of the Day	End of the Night	Degradation Rate
**Col-0**	R	36.7 ± 4.3^AD^	2.3 ± 0.5^a^	93.7%
***dpe2/phs1***	P	47.4 ± 9.0^A^	19.8 ± 4.0^b^	58.2%
	Y	77.3 ± 2.5^B^	56.0 ± 5.5^c^	27.6%
	M	19.9 ± 4.8^C^	14.3 ± 3.6^b^	28.1%
	R	23.4 ± 6.4^CD^	18.1 ± 3.0^b^	22.6%

Leaves were harvested at the end of the light and dark phase. Mean values ± SD are given, letters indicate the statistically significant differences (p ≤ 0.05) according to the one-way ANOVA with Duncan’s post-hoc test.

P, Prior Period; Y, young leaves of Reduced Granule Number Period; M, mature leaves of Reduced Granule Number Period; R, Recovery Period.

Scanning electron microscopy (SEM) revealed that the starch granule morphology was strongly altered in *dpe2/phs1* in all three phases (**Figures S2A, B**). Col-0 leaves at the same age as the individual phases of mutant were harvested; however, since there was no evident difference detected among aging materials of Col-0, only one data is presented here. *Dpe2/phs1* showed the largest long axis and thickness in young leaves in RGN (**Figure S2B**). Moreover, in consistent with the LCSM, R had the most inhomogeneous size distribution, with more tiny granules, which could be related to newly generated granules in the recovered chloroplasts. Regarding the inner starch structure, the mutant revealed increased amounts of glucan chains with DP 16-18, accompanied by decreased amounts of shorter chains (DP 5-10) in the amylopectin, compared to Col-0 in all three stages (**Figure S2C**). The changes in the inner starch structure intensified with the age of the mutants.

### Gene expression differed most in young and mature leaves of RGN

One way to investigate the reasons underlying the variation in the starch granules through the three phases is to analyze the associated gene expression by RNA-seq. Therefore, leaves from P, where the chloroplasts contained the normal starch granule number; young Y and mature M leaves from RGN, where most of the chloroplasts had only a single, large, and round starch granule and most chloroplasts were aberrant, respectively; and leaves from R, where the chloroplasts and starches recovered, were analyzed. As a control, Col-0 samples of the same age were used.

**Figure 3:**
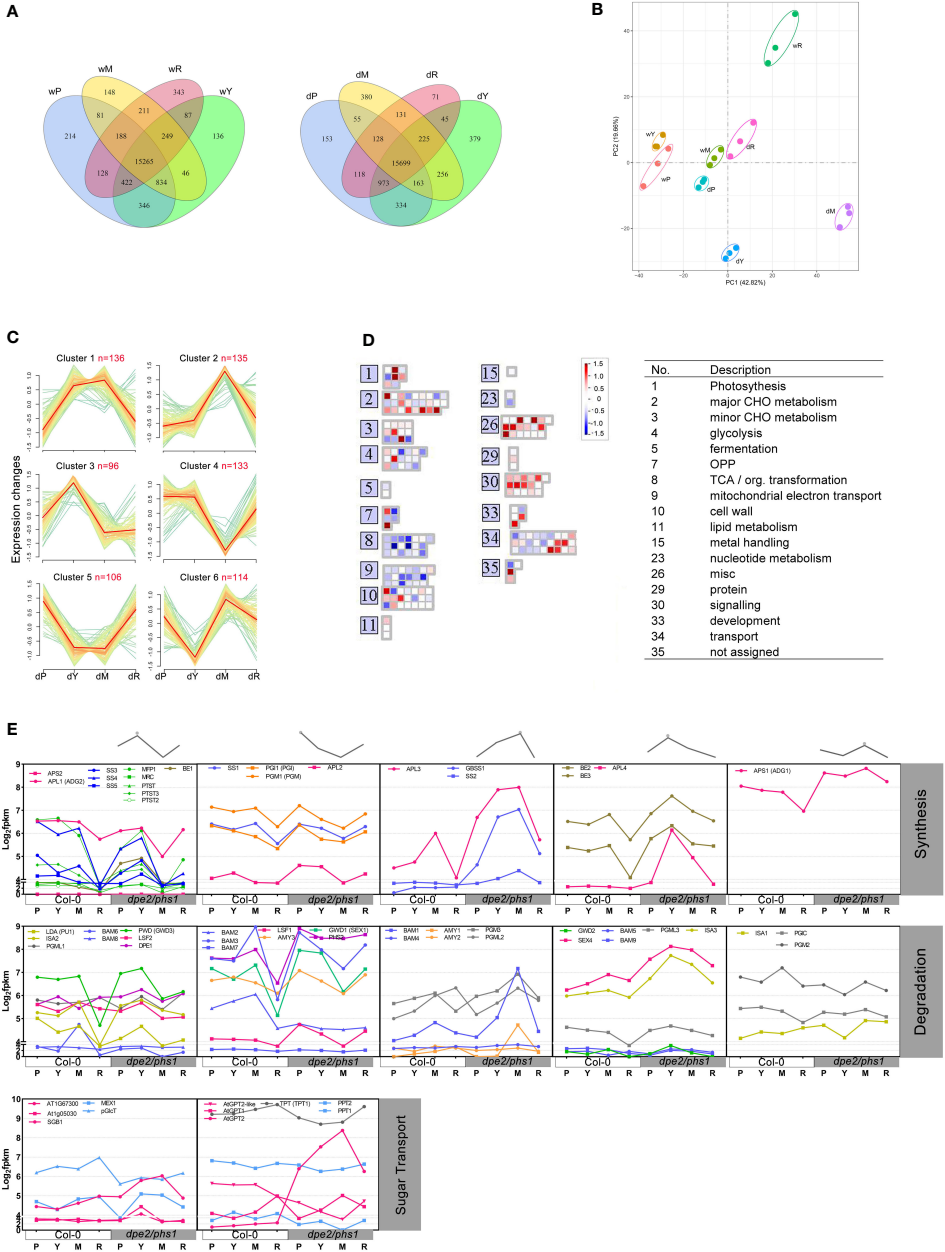
Differential gene expression in *dpe2/phs1* and Col-0. w, wild-type; d, double mutant *dpe2/phs1*: **(A)** Venn diagrams showing the number of significantly differentially expressed genes among the three periods, based on the cut-off of adjusted *P* value < 0.05. Young (Y) and mature leaves (M) in the Reduced Granule Number Period were analyzed separately; **(B)** PCA plot in different periods for *dpe2/phs1* and Col-0. **(C)** Differential expression of 720 genes from nine pathways were assigned to six clusters. Each gene was normalized according to log_2_(FPKM+1). Each green line represents the expression level of a particular gene relative to the median expression level of all genes in that expression category. Orange lines represent the median expression; **(D)** Differential expression level of genes in clusters 3 and 6 were visualized with MAPMAN (Thimm et al., 2004). The differences between dY relative to the wY and dP relative to wP are shown in colors (blue, low expression; red, high expression); **(E)** Clustered starch synthesis, degradation, and sugar transport genes (log_2_(FPKM+1)). The grey lines at the top represent the gene expression pattern, which is only based on the trend in *dpe2/phs1*. The synthesis and degradation enzymes in the same column shared the same pattern. The abbreviations for the starch metabolism-related genes are given in **Table S1**.

Comparison of the differentially expressed genes using the R package DESeq2 (adjusted *P*-value < 0.05) revealed only a moderate number of genes specifically expressed in the various phases (**Figure 3A**). In Col-0, most genes uniquely altered in expression were found in R, which were likely linked to the initiation/generation of florescence. *Dpe2/phs1* revealed genes expressed differently compared with Col-0. The highest number of genes was found, as expected, in young and mature leaves of RGN (379 and 380, respectively; **Figure 3A**). Principal component analysis revealed that, besides R of Col-0, both Y and M of *dpe2/phs1* were clustered away from the other groups (**Figure 3B**). The separation of R from Col-0 is probably related, as mentioned above, to flowering. Interestingly, the R of *dpe2/phs1* did not separate, likely due to the associated delay in development (**Figure 2**).

### Transcript level alterations of most starch metabolism related genes are clustered in the three phases of *dpe2/phs1*

Furthermore, an H-cluster analysis of the *dpe2/phs1* groups was performed, including the genes annotated to nine metabolic pathways: metabolism of starch and sucrose (pathway ID: ath00500), galactose (ath00052), fructose and mannose (ath00051), pentose phosphate (ath00030), pentose and glucuronate interconversions (ath00040), inositol phosphate (ath00562), citrate cycle (ath00020), glycolysis (ath00010), and oxidative phosphorylation (ath00190). Overall, 720 genes were assigned to six clusters (**Figure 3C**). The results revealed that, following RGN, the gene expression also recovered; thus the expression of genes in P and R was highly similar. This was also supported by Heatmap analysis (**Figure S3**). Clusters 1, 2, and 6 had the relative highest expression levels in M, whereas cluster 4 had the lowest. Cluster 3 had the fewest enriched genes (96), and it was the only cluster that expressed relatively high in Y. Regarding the correlation with the starch granule number per chloroplast, clusters 3 and 6 were most interesting, as they showed the strongest alteration in Y; that is, when the starch granule number was reduced (**Figure 1**). MAPMAN was used to investigate 210 genes from those clusters (**Figure 3D**) (Thimm et al., 2004). The change in expression between Y and P was analyzed. TCA and mitochondrial electron transport genes were dramatically reduced. The genes related to signaling also uniformly increased in expression. However, genes related to major carbohydrate metabolism were differentially affected.

### High expression of genes involved in starch degradation was observed in young leaves of RGN that showed a decreased starch granule number

In young leaves of RGN, the starch granule number per chloroplast decreased to one, thus an uneven degradation rate occurred on the original three to seven granules, and the disappeared granules were totally degraded. Therefore, it is rational to suppose higher expression levels of starch degradation-related genes. The differential expression of genes involved in starch metabolism in both Col-0 and *dpe2/phs1* were analyzed (**Figure 3E**). Obviously, genes were co-regulated in different clusters, similar to that shown in **Figure 3C**. Most starch degradation genes (20 in total), revealed an increase in Y and nearly similar values between P and R. However, 10 genes including BAM3, AMY3, ISA1, and GWD1 showed a decrease in Y comparing to P and R.

Interestingly, most genes involved in starch initiation, including SS4, SS3, PTST, PTST2, PTST3, MFP1, and MRC, also revealed a higher expression in Y (Merida and Fettke, 2021). Considering the higher amount of starch content in young leaves and only one existing starch granule, it is possible that the sugars released from the other granules were recycled to synthesize the existing single starch granule in Y. This is also supported by the lower sucrose content of *dpe2/phs1* (Malinova et al., 2014). The higher expression level of genes involved in synthesis may reflect an attempt to overcome the limitation in starch granule number observed in young leaves in RGN.

Moreover, the homologous genes did not follow the same clustering; for instance, starch synthases SS2–5 and GBSS1 increased from P to young leaves in RGN, but not SS1. However, the observed alteration was in agreement with the chain length distribution profile of the starch granules, as fewer short chains (related to SS1) and more medium–long chains (related to SS2) were observed (**Figure S2C**). Among the transporters, GPT2 showed the most significant alteration through the four stages. The RNA-seq results were validated through the qRT-PCR of eight example genes (**Figure S4**).

### The observed phenotypical periods of *dpe2/phs1* are perpetuated when starch degradation is partially impaired

**Figure 4:**
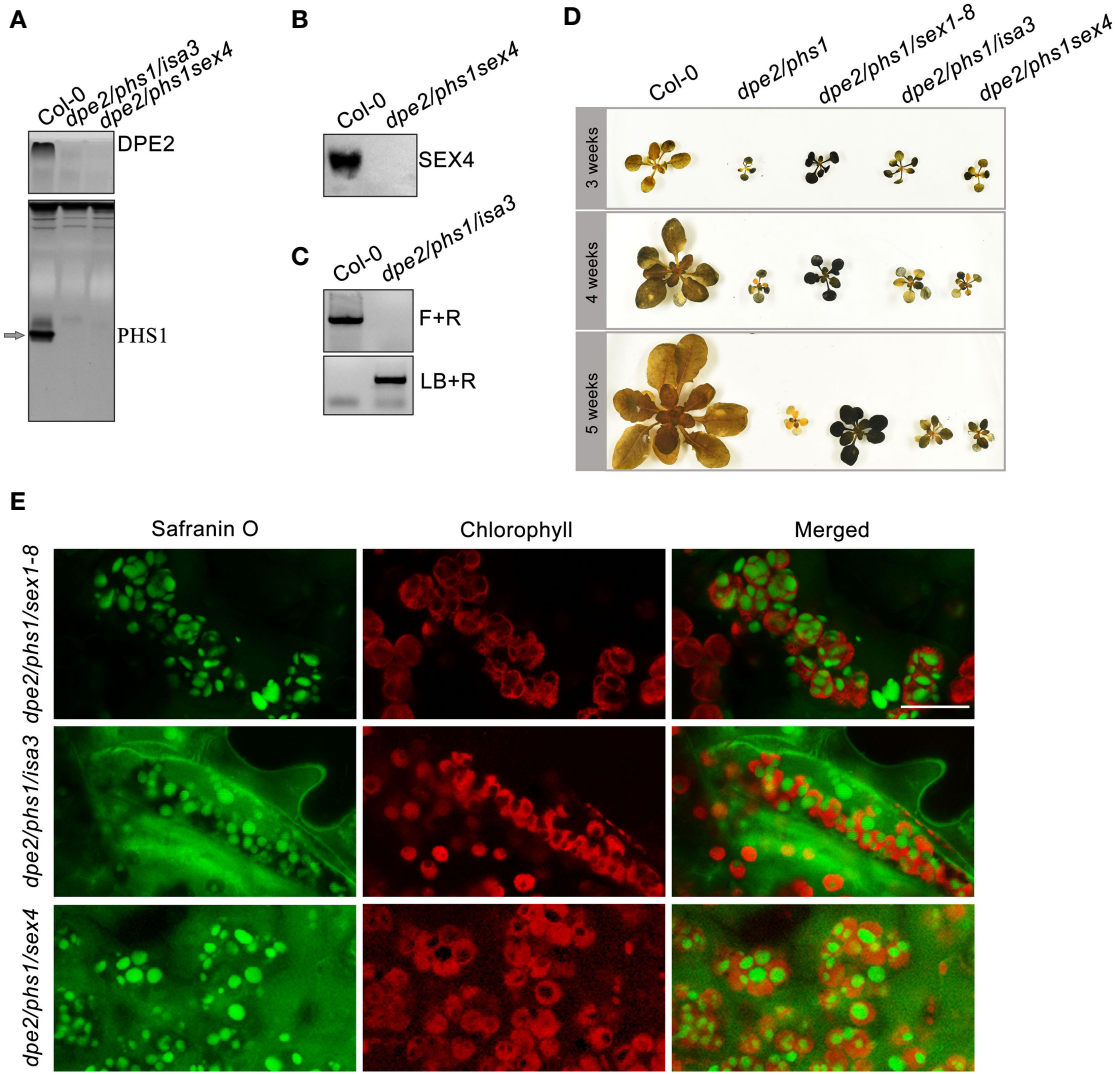
The three distinguishable periods observed in *dpe2/phs1/sex4* and *dpe2/phs1/isa3* grown under light/dark regime: **(A)** Confirmation of the lack of activity of PHS1 and DPE2 by native PAGE. The arrow indicates the position of PHS1; **(B)** Detection of SEX4 by Western blot and immunodetection. A total of 10 μg protein was loaded per lane for analysis in A and B; **(C)** T-DNA insertion of ISA3. Forward **(F)** and Reverse (R) primers were used to check homozygosity. R and LB o8474 (LB) were used to check T-DNA insertion; **(D)** Semi-quantitative starch determination from the three periods. Shoots were harvested at the end of the light phase at three, four, and five weeks, respectively. **(E)** LCSM observation of starch granule number per chloroplast in Y of the triple mutants, leaves were taken from 3-week plant as shown in **(D)** Bar = 10 µm.

To verify whether starch degradation-related genes which had higher expression level in Y were involved in granule number reduction, the additional knockout of these genes in *dpe2/phs1* background was necessary. New triple mutants were generated by crossing *dpe2/phs1* with single mutants lacking Starch excess 4 (SEX4; EC: 3.1.3), an important phosphatase in the phosphorylation/dephosphorylation cycle at the starch granule surface, and Isoamylase 3 (ISA3; EC:3.2.1.68), a hydrolase involved in starch degradation, both genes revealed an increase in Y. Both triple mutants revealed reduced shoot growth and uneven starch distribution, in addition, a decreased starch granule number, similar to that of *dpe2/phs1* rather than Col-0 (**Figure 4**). Further, the newly generated triple mutants revealed similar phenotypical alterations during growth, while they overcame the dysfunctional RGN stage quickly, as the triple mutants recovered in the fifth week while *dpe2/phs1* reached its RGN. Similarly, it has been found after knock-out of Phosphoglucan, water dikinase (PWD) and Disproportionating enzyme 1 (DPE1; [EC: 2.4.1.25]) it was not possible to fully recover the wild-type starch phenotype in the background of the *dpe2/phs1* mutant (Muntaha et al., 2022). In contrast, the additional lack of α-Glucan, water dikinase (GWD), a key enzyme in the starch phosphorylation/dephosphorylation cycle prior to starch degradation (Ritte et al., 2006; Edner et al., 2007; Hejazi et al., 2012), resulted in normal starch granule numbers in young and mature leaves (Malinova and Fettke, 2017), and *dpe2/phs1/sex1-8* did not present three distinguishable periods (**Figure 4**). Thus, different influences of the varying extent of ongoing starch degradation on the developmental phenotype of *dpe2/phs1* were observed. An additional lack of SEX4, PWD, ISA3, and DPE1, all these enzymes act downstream of GWD, result still in an altered starch granule number in Y, therefore an even partially ongoing starch degradation seems essential for the development of the observed phenotypic features.

### Basal transcription factors, MAPK signaling, and galactose metabolism were significantly enriched in young leaves in RGN, when the starch granule number is massively reduced

**Figure 5:**
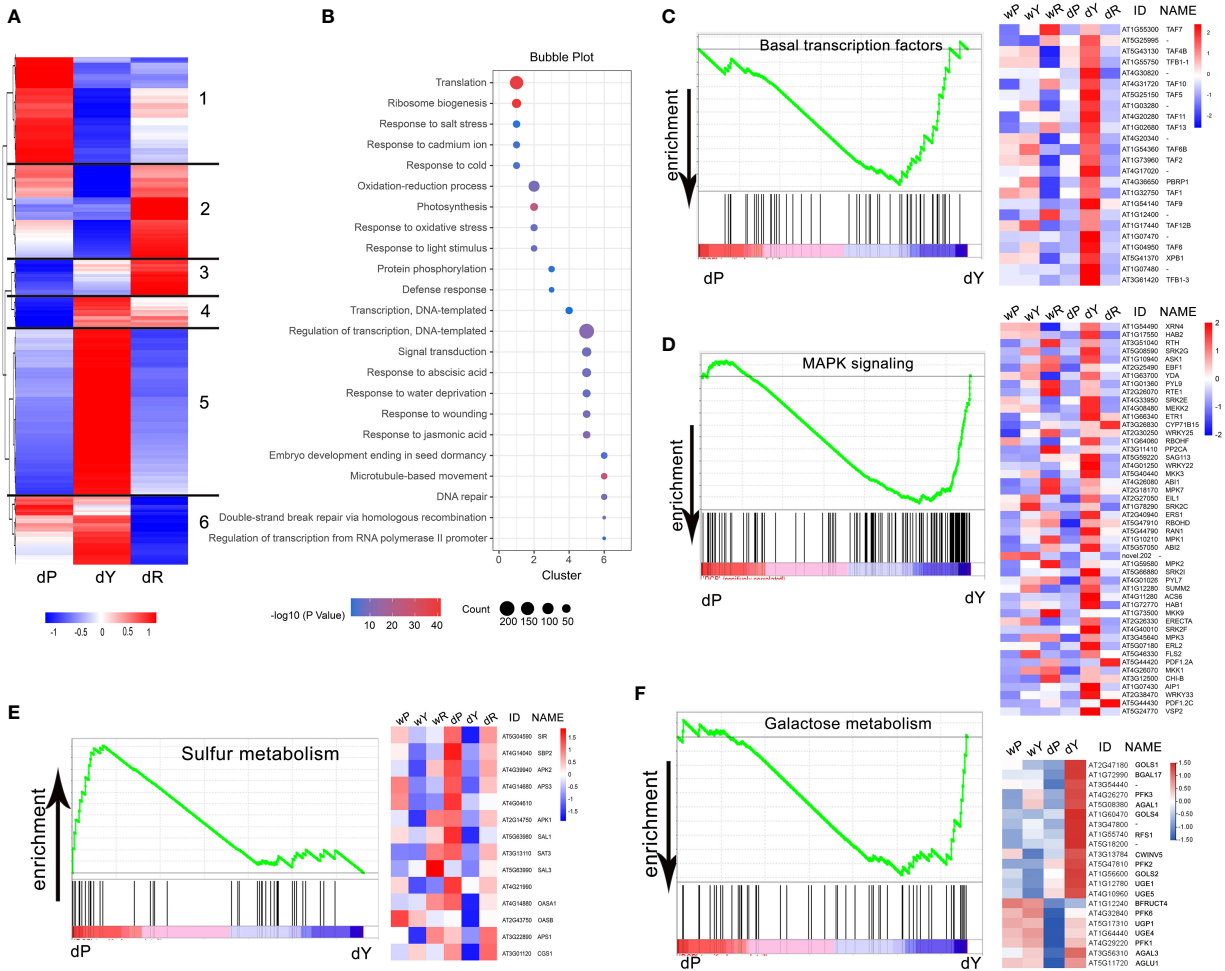
Enrichment analysis of differentially expressed genes in P, Y, and R: **(A)** Heatmap representation of 5517 genes differentially regulated in P, Y, and R (FC > 2, adjusted *P* value < 0.01). Six clusters of co-regulated genes are indicated. Color pattern represents row Z-score; **(B)** Bubble chart showing results of Gene Ontology (GO) enrichment analysis. Bubble size indicates number of genes associated with each term. Bubble color indicates −log_10_(*P* value); **(C, D, F)** GSEA plots for pathways enriched in dY compared to dP (FDR < 25%). Selected gene expressions are presented in heatmaps, the gene expressions in R are also shown in **(C, D)**; **(E)** GSEA plot for pathways enriched in dP compared to dY (FDR < 25%), selected gene expressions are presented in heatmaps, the gene expressions in R are also shown.

To further investigate the differentially regulated pathways involved in the reduced starch granule number, we performed gene enrichment analysis by comparing P with Y, and Y with R, respectively. According to the cut-off of FC > 2 and adjusted *P*-value < 0.01, 5517 genes were filtered. Hierarchical clustering showed at least six distinct clusters of co-regulated genes (**Figure 5A**). Genes enriched in clusters 1 and 2 were expressed relatively lower in Y, while an opposite expression trend was observed in P and R. Gene Ontology enrichment analysis revealed that cluster 1 included genes involved in translation and ribosome biogenesis, while cluster 2 included genes relevant to oxidation–reduction processes and responses to light stimulus, which may be linked to the observed recovery of chloroplasts (and, consequently, photosynthesis) (**Figure 5B**). The genes in cluster 3 showed a moderate expression level in Y, which were related to protein phosphorylation and defense response (**Figure 5B**). In contrast, clusters 4, 5, and 6 included genes that were strongly increased in Y but expressed differentially in P and R. Cluster 5 contained the most genes. Most of the enriched genes in this cluster were involved in stress responses, which was also expected from the observed phenotype.

To identify pathways enriched in Y in RGN, we performed gene set enrichment analysis (GSEA) by comparing P with Y, and Y with R, respectively. GSEA between P and Y showed that 111 gene sets were enriched, with 62 sets up-regulated and 49 sets down-regulated in P. Nine gene sets were significant at FDR < 25% (**Table S2**). Only sulfur metabolism was significantly enriched in P, while basal transcription factors, MAPK signaling, and galactose were significantly enriched in Y. No significantly affected pathway was found between Y and R at FDR < 25% (**Table S3**). Basal transcription factors, MAPK signaling enriched in Y, and sulfur metabolism enriched in P were significant, with nominal P-value < 0.01. For selected genes, the expression levels are shown (using heatmaps) in **Figures 5C–F**.

### Disintegrated chloroplasts in mature leaves in RGN

**Figure 6:**
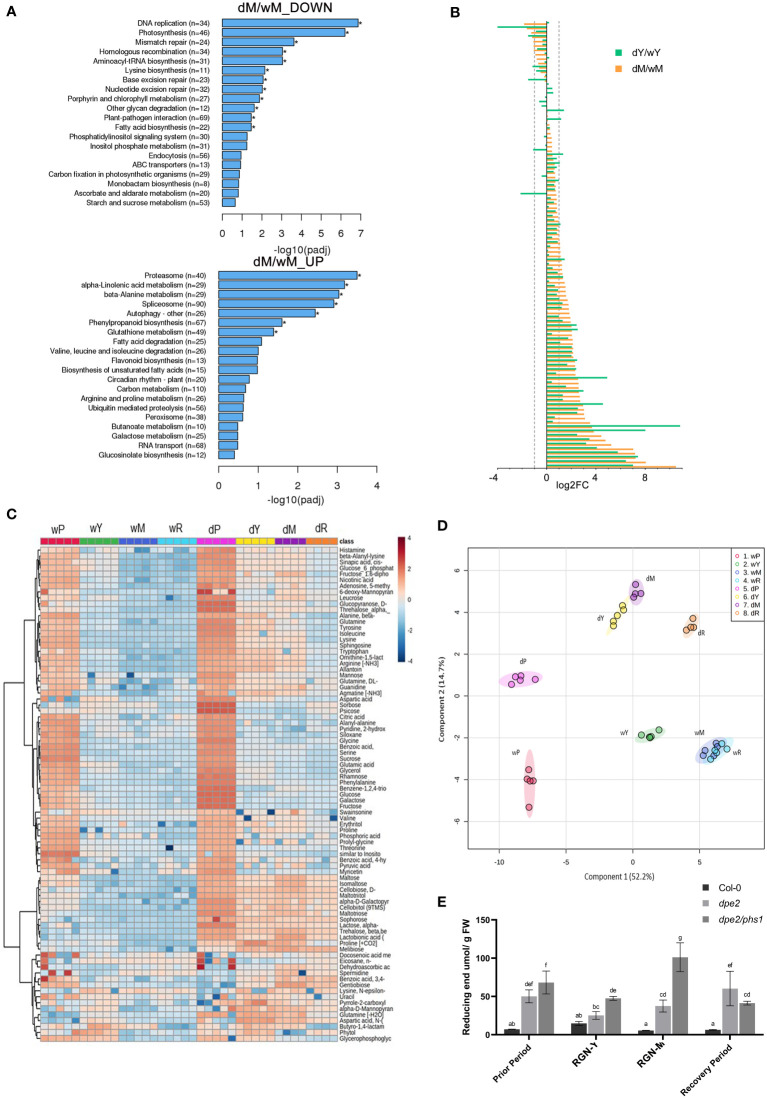
Significantly differentially regulated pathways in *dpe2/phs1* mature leaves and metabolic profiling: **(A)** KEGG bar charts indicating the pathways differentially regulated between *dpe2/phs1* and Col-0 mature leaves; **(B)** Changes in expression of 105 senescence-related genes. The difference between the young leaves of *dpe2/phs1* compared to age-matched leaves of Col-0 (dY/wY), and the mature leaves of *dpe2/phs1* compared to age-matched leaves of Col-0 (dM/wM) are shown. **(C)** Heatmap analysis showing selected metabolites; **(D)** Partial least squares discriminant analysis (PLS-DA) results showing the sample clusters. A minimum of four biological replicates is presented for each. Values are expressed as concentrations normalized to median values; **(E)** Maltodextrin content. All values are mean ± SD (n = 3 replicates from a mixture of 8–10 plants each harvested after 9 hours of illumination). Letters indicate statistically significant differences (p ≤ 0.05) according to one-way ANOVA with Duncan’s *post-hoc* test.

Most chloroplasts in mature leaves in RGN were aberrant and starch-free (**Figure 1**), which was related to the obvious chloroplast degradation. Thus, the up- and down-regulated genes in Clusters 2 and 4 (**Figure 3C**) were most interesting, with regard to the observed chloroplast intactness. A similar phenotype with chlorotic leaves has been reported for *mex1* mature leaves (Stettler et al., 2009), although the yellowing was moderate, compared to that of *dpe2/phs1* (**Figure 2** *)*. In addition, *mex1* was not completely devoid of starch. Chloroplast autophagy has been reported to be linked to the leaf senescence. In comparison to Col-0 leaves of the same age, the autophagy and protease pathway were significantly up-regulated in the mature leaves of *dpe2/phs1* (**Figure 6A**). Meanwhile, fundamental activities, such as DNA replication, mismatch repair, and photosynthesis, were dramatically down-regulated (**Figure 6A**). It has also been reported that most senescence-induced genes changed less than 2-fold in *mex1*, in comparison to Col-0 (Stettler et al., 2009). To investigate the difference between Y and M, we compared the expression of 105 genes between Y and the Col-0 control (dY/wY), and M and the respective Col-0 values (dM/wM), respectively (**Figure 6B**). In mature leaves in RGN, more up-regulated genes relative to the situation in young leaves were detected, as expected from the phenotypical characterization. A total of 98 genes were up-regulated in mature leaves and 53 genes changed more than twice, including the genes: autophagy 8C (ATG8C, AT1G62040), ATG8D (AT2G05630), autophagy 8E (AT2G45170). In young leaves, 80 genes were up-regulated and only 39 of them changed more than twice. However, the senescence-associated gene 21 (SAG21, AT4G02380) up-regulated more than twice both in Y and M, which were 6 and 12 times up-regulated, respectively.

### Metabolic analysis revealed alterations in general metabolism in the three phases of *dpe2/phs1*

A total of 92 metabolites were determined, which are presented in a heatmap in **Figure 6C**. Partial least squares (PLS) analysis revealed that the various observed periods were metabolically different, but no outstanding alteration in RGN is given (**Figure 6D**). This obviously differed from the described phenotypic and transcriptomic alterations. However, no clear separation was detected for M and R in the wild-type samples, which was not unexpected as no altered periods were observed in the wild-type, and the leaf material was only separated according to *dpe2/phs1*.

A detailed look at the carbohydrate-related metabolites revealed that sugars related to starch degradation, such as maltose, maltotriose, isomaltose, and trehalose, showed significantly higher levels in *dpe2/phs1*, compared to the wild-type, in all periods (**Figure 6C**). To investigate the effect on maltodextrins, we also analyzed the maltodextrin content in the three periods (**Figure 6E**). In all periods, except in R, *dpe2/phs1* accumulated more maltodextrins compared to both the wild-type and the *dpe2* parental line. The highest accumulation was, indeed, observed in the mature leaves of RGN. However, no distinct DP of the maltodextrins was affected in RGN; instead, an accumulation of maltodextrins and longer chains (DP 10, 11 and 12) were observed in all periods in *dpe2/phs1*, except a lower DP 11 in R (**Figure S5**). Thus, PHS1 seems to be involved in the depolymerization of longer maltodextrins (DP longer 9), which is impossible in *dpe2/phs1*. Also, even less likely, the accumulation of maltodextrins could be a result of missing further extension of the glucans by the lack of the enzyme.

## Discussion

This study was inspired by a detailed investigation of *dpe2/phs1* using a rapid method (LCSM combined with Safranin O; Liu et al., 2021), allowing for determination of the starch granules in leaves in different periods. Previously, *dpe2/phs1* has been reported as having a severely impaired growth rate and heterogeneous starch distribution, with a single starch granule per chloroplast in young leaves and almost starch-less mature leaves when grown under diurnal rhythm. Our investigation revealed that starch metabolism in *dpe2/phs1* is inhomogeneous not only among leaves, but also during development. Thus, three clearly distinguishable periods were described. By analyzing relevant leaf material from these three stages through RNA-seq and metabolic assays, we determined the co-regulated genes involved in starch metabolism, showing a clearly separated metabolism, suggesting a regulation of the starch granule number throughout the plant growth. This unique plant material, thus genetically identical—as is not the case when working with mutants—allowed us to understand the important step within the starch metabolism, regulation, and initiation of starch granules in Arabidopsis leaves.

When grown under continuous light, *dpe2/phs1* was homogeneous in both starch distribution and starch granule number over the entire growth period (**Figure S6**; Malinova et al., 2014).

### Starch granule number varied in three periods of *dpe2/phs1*

Chloroplasts in P revealed the typical starch granule number observed in the wild-type, with 1–7 granules per chloroplast (Smith et al., 2005; Stitt and Zeeman, 2012); **Figure 1**). However, a slight shift to RGN per chloroplast was already observed in *dpe2/phs1* in this early state, pointing to a progressive alteration. The shoot iodine staining results revealed the homogeneous starch distribution (**Figure 2A**) and also that the starch in root caps was normal (**Figure 2C**), indicating that the starch metabolism was less affected and similar to the situation in the wild-type.

Different from P, RGN revealed the most reduced growth rate both in shoots and roots, with nearly no new formed leaves and deformed root caps. Consistent with previous reports (Malinova et al., 2014), this period mostly revealed only a single starch granule per chloroplast in young leaves, while starch-less and aberrant chloroplasts were observed in mature leaves (**Figure 1**). Given the strongly reduced growth rate and the absence of newly formed leaves during this period, the existence of a single granule is more likely the product of granule reduction in the chloroplasts, rather than an initiation failure. Thus, *dpe2/phs1* lost its ability to maintain a normal starch granule number. Thus, the former interpretation that *dpe2/phs1* affects the initiation of starch granules in leaves is not correct (Malinova et al., 2014), as *dpe2/phs1* can generate the normal starch granule number per chloroplast, but lost control over that number during development. Considering the reduced starch granule number, the increased expression of genes involved in starch degradation in Y was expected. Thus, RNA-seq results indicated that 20 of the degradation related genes increased meanwhile 10 decreased. However, the knockout validation of SEX4 and ISA3 in *dpe2/phs1* background still showed the reduced granule number and growth periods. Moreover, the observed single starch granule per chloroplast indicated an uneven starch metabolism inside the chloroplasts. The starch content in Y was higher than that in Col-0 (**Table 1**), which points to the fact that the single granule was further used for synthesis, while the rest of the granules were totally degraded. Thus, the action of enzymes involved in starch metabolism have to differ on the surfaces of the various starch granules inside of a chloroplast. However, how this granule is defined and whether it reveals specific characteristics remain obscure. Interestingly, in the last period, it recovered control and, thus, the granule number increased, although the number was still decreased compared to that in the wild-type. This also indicates that the granule number is controlled throughout the growth of the plant, not only once initiated and then kept constant. It could be that the starch granules were not totally depleted at dawn and, so, the remaining granules were reused for synthesis in wild type (Burgy et al., 2021). This is unlikely the possibility here, as we observed not a more intense but, instead, an impaired starch degradation, allowing the removal of the initiated starch granules prior to starch synthesis. In contrast, *dpe2/phs1* revealed a reduced starch degradation (**Table 1**). Thus, an independent control mechanism exists, which is affected in *dpe2/phs1*, allowing for control over the starch granule number per chloroplast. Interestingly, when the starch degradation was severely impaired, in the *dpe2/phs1/sex1-8* triple mutant a similar starch granule number per chloroplast compared to wild-type was observed (Malinova and Fettke, 2017); pointing to the effect of starch degradation on starch granule number regulation. Further, GWD may be involved in regulation of the starch granule number. This would also explain why, in sex1-8, not only starch synthesis, degradation, and morphology, but also the granule number per chloroplast is affected (Mahlow et al., 2016). That not the starch degradation *per se* is important for granule number regulation is obvious when comparing the granule number per chloroplast in *dpe2/phs1/pwd* and *dpe2/phs/dpe1*. In these mutants the granule number was reduced, compared to the wild-type, even when starch degradation was strongly impaired (Muntaha et al., 2022). Thus, either the total blocking of starch degradation, mediated by a lack of GWD, or the lack of GWD *per se* may be responsible for the observed starch granule number regulation. Interestingly, the transcript data revealed that GWD transcript level decreased during RGN from young to mature leaves but increased massively in R (**Figure 3E**), similarly to the starch granule number. However, this evidence is weak, as other proteins involved in starch degradation, such as BAM2, BAM3, and AMY3, also revealed the same trend (**Figure 3E**). However, these degrading enzymes may be involved in consuming the rest of the starch granules totally. In contrast, PWD and others did not show such clustering (**Figure 3E**).

So far unknown, but also interesting, is whether other mutants with affected starch granule number also display different periods regarding the starch granule number per chloroplast and, if so, are they comparable or not? Answering these questions may allow us to obtain even more insights into the regulation of starch granule number per chloroplast.

The SEM results for isolated native starch granules were consistent with the LCSM observations (**Figures 1** and **S2**). The young leaves in RGN contained the largest and roundest starch granules. A single large spherical granule was frequently found in R, most likely inherited from RGN (**Figure S2**), which points to additional starch granule initiation in R and unequal starch granule growth. Thus, the normal control mechanisms for the more-or-less uniform starch granules were affected.

Similar to the phenotypic differences, the RNA-seq results clearly revealed that the young and mature leaves in RGN of *dpe2/phs1* were separated from the rest. Comparing the gene expressions in three periods, we clearly showed the co-regulation of genes involved in starch metabolism, which clustered differently in the three stages (**Figures 3C, E**).

### Chloroplast stability in *dpe2/phs1*

In R, both the starch granule number and the starch distribution were recovered, indicating an obvious re-genesis or repair of chloroplasts; however, it remains obscure how this recovery is regulated. The chloroplasts, as the energy supply center where photosynthesis takes place and starch is stored, are vital for fundamental plant activities, and it is highly likely that, when most chloroplasts are corrupted, a signal is transduced that induces the re-construction/re-genesis of chloroplasts to overcome energy shortages. However, a DNA mutation in nucleus can be excluded, as the next generations of *dpe2/phs1* also presented the same three periods.

It has been postulated that there exists a connection between chloroplast degradation and accumulated maltodextrins (Stettler et al., 2009). Here, the very high maltodextrin content observed in the mature leaves of the mutant support this idea (**Figure 6E**); however, the monosaccharide content in mature leaves in RGN of *dpe2/phs1* was moderate, indicating that the longer maltodextrins, rather than the monosaccharides, were the key factor affecting chloroplast stability. Similarly, a relatively high maltodextrin level was detected in young leaves in RGN which contained a single granule per chloroplast. In comparison to the young leaves, the mature leaves accumulated much more maltodextrins (**Figure 6E**), thus following the idea that a higher expression level of senescence- or autophagy-related genes was expected. This was confirmed by RNA-seq (**Figures 6A, B**). Also here, most senescence-induced genes were highly expressed in mature leaves, with many of them being increased more than twice. Thus, our results differed from those described for the *mex1* mutant, in which most relevant genes changed less than twice (Stettler et al., 2009). Therefore, a less severe degradation of chloroplasts occurs in *mex1*.

Further, decreased maltodextrin accumulation (*via e.g*., blocking of starch degradation) may reduce chloroplast degradation. Thus, it has been reported that the total blocking of starch degradation due to a lack of GWD resulted in the complete restoration of chloroplasts (Malinova and Fettke, 2017). In contrast, under the partial blocking of degradation due to a lack of PWD and DPE1, chloroplast disintegration still occurred (Muntaha et al., 2022). Partial blocking of starch degradation *via* a loss of SEX4 and ISA3 in this study also resulted in starch-less mature leaves (**Figure 3**).

On the other hand, it may also be that the longer DPs observed in *dpe2/phs1* (**Figure S5**) are normally used during starch initiation, which does not happen here, thus the maltodextrins were not used for starch initiation, resulting in a reduced starch granule number per chloroplast in *dpe2/phs1* independently of the period (**Figure 1**).

In this paper, we presented the unique phenotype of *dpe2/phs1*, which lose the control over starch granule number regulation during growth. RNA-seq results revealed that most of the degradation involved genes increased when starch granule number decreased to one in Y. However, triple mutants lacking SEX4 or ISA3 in the background of *dpe2/phs1* were unable to recover the reduction in starch granule number. In contrast, the additional lack of GWD results in a recovered starch granule number. The different ability to recover the starch granule numbers in these triple mutants indicate the different influences of the varying extent of ongoing starch degradation on the starch granule number. Further, the different starch granule numbers during the development reveal a metabolic control of the granule number and not a simple genetic regulation. Overall, *dpe2/phs1* is a promising plant source for understanding control of the starch granule number per chloroplast throughout the plant development, which can shed light on the regulation of chloroplast stability; at least, in mutants with affected starch metabolism.

## Data availability statement

The datasets presented in this study can be found in online repositories. The names of the repository/repositories and accession number(s) can be found in the article/**Supplementary Material**.

## Author contributions

XL and JF conceived and designed the experiments; XL, AA, JFC, JC, and SM conducted the experiments; XL and JF wrote the manuscript; JF revised the manuscript. All authors read and approved the final manuscript.

## Funding

This work was supported by the Deutsche Forschungsgemeinschaft DFG-FE 1030/5-1, and 6-1.

## Acknowledgments

This research was further supported by INST 336/114-1 FUGG for large equipment, granted to Prof. Markus Grebe and co-applicants.

## Conflict of interest

The authors declare that the research was conducted in the absence of any commercial or financial relationships that could be construed as a potential conflict of interest.

## Publisher’s note

All claims expressed in this article are solely those of the authors and do not necessarily represent those of their affiliated organizations, or those of the publisher, the editors and the reviewers. Any product that may be evaluated in this article, or claim that may be made by its manufacturer, is not guaranteed or endorsed by the publisher.
