# Supplementary material for: Dpe2/phs1 revealed unique starch metabolism with three distinct phases characterized by different starch granule numbers per chloroplast, allowing insights into the control mechanism of granule number regulation by gene co-regulation and metabolic profiling
Source: Front Plant Sci. 2022 Nov 1;13:1039534. doi: 10.3389/fpls.2022.1039534 (PMC9667719; doi:10.3389/fpls.2022.1039534)
Supplement: Supplementary file 1 [file DataSheet_1.pdf]

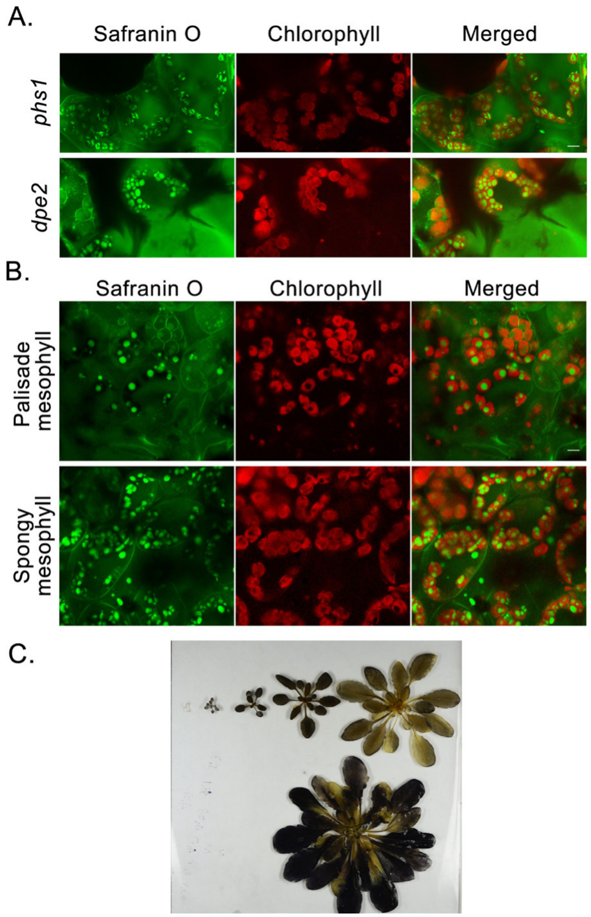

Figure S1 LCSM analysis of starch granules per chloroplast. (**A**) Both *phs1* and *dpe2* showed normal starch granule numbers per chloroplast. (**B**) LCSM analysis of *dpe2/phs1* within the same leaf between the Prior and Reduced Granule Number Periods (RGN). Leaves were taken from four-week-old rosettes in the middle of the light period. Bar = 5 μm.(C) Semi-quantitative starch determination of Col-0. Shoots harvested at the EOD from two to seven weeks, decolorized shoots were stained with iodine solution.

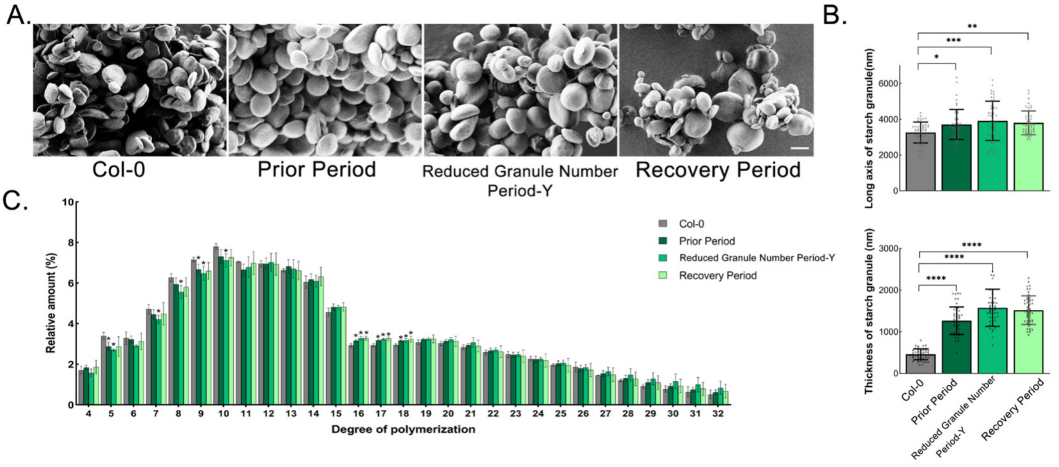

Figure S2 Starch granule characteristics of Col-0 and the different periods of *dpe2/phs1*: (**A**) SEM analysis. Starch granules were isolated from leaves harvested at the end of the light phase. Bars = 2 μm; (**B**) Long axis and thickness of starch granules. Mean values and SD are shown, n = 50; (**C**) Chain length distribution of starch granules. All values are mean ± SD (n = 3). Asterisks show the significant differences between Col-0 and the different periods of *dpe2/phs1*: *, $p < 0.05$; **, $p < 0.01$; and ***, $p < 0.001$; based on t-tests for both B and C.

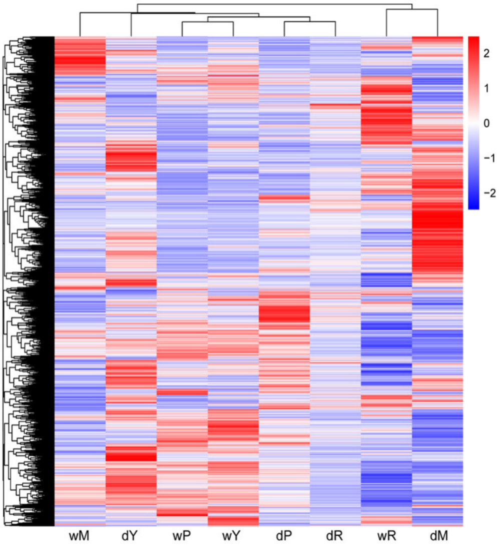

Figure S3 Heatmap plot of differential expression in the three periods for *dpe2/phs1* and the wild-type control, respectively. Heatmap shows significantly (adjusted *P* value < 0.05) differentially expressed genes across eight groups. Differentially expressed gene values in each row were transformed according to $\log_2$(FPKM+1) (blue, low expression; red, high expression). W, wild-type; d, double mutant *dpe2/phs1*.

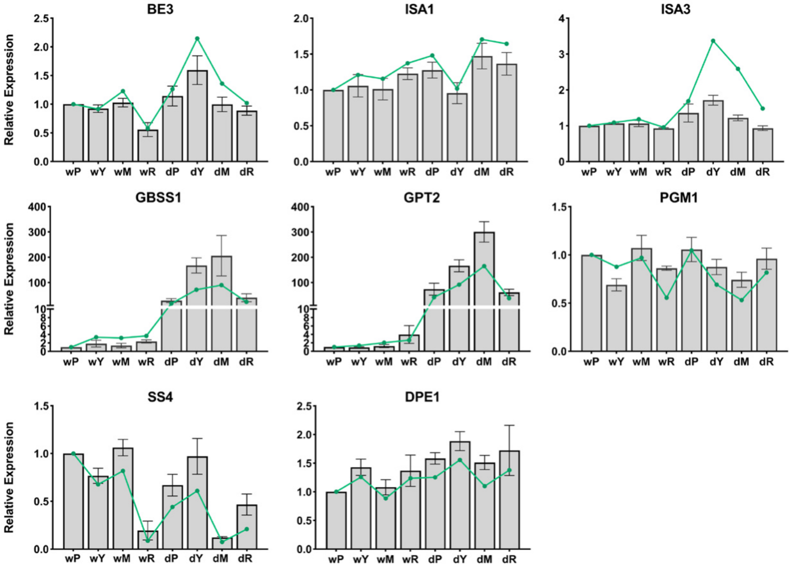

Figure S4 qRT-PCR validation of the expression levels of eight starch-related genes. The comparison between RNA-Seq data (grey bar) and qRT-PCR data (green line) is shown. Mean values ± SD of three replicates are shown in the qRT-PCR data. Actin was used as an internal control. The RNA-Seq results were normalized based on log_2FPKM. Both methods agreed with each other, showing similar gene expression tendencies. The abbreviations for the starch metabolism-related genes are given in Table S1.

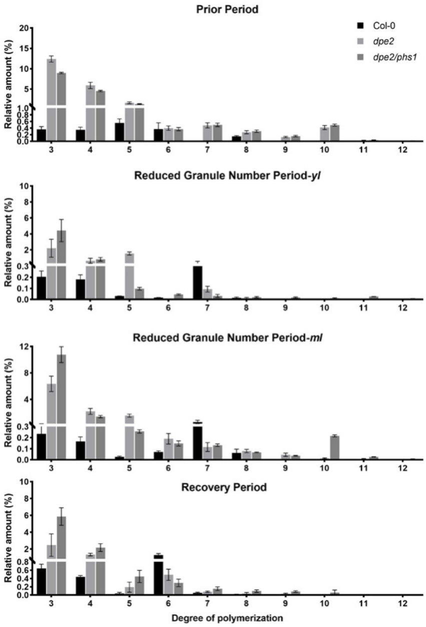

Figure S5 Relative amount of maltodextrin in the three periods. Rosettes were harvested at 3, 5, and 6 weeks, respectively. CE-LIF was used to analyze the relative amount of maltodextrin.

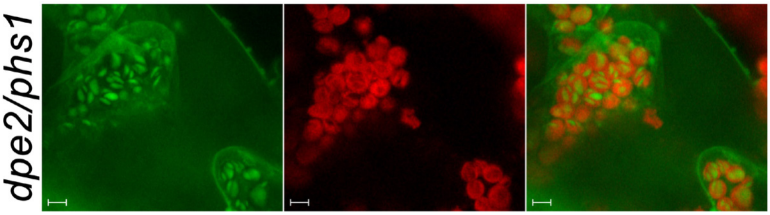

Figure S6 LCSM analysis of starch granules in chloroplasts of *dpe2/phs1* grown under continuous light. Leaves were taken from 2-week-old rosettes of the mutant. Bar = 5 µm.

Table S1 The abbreviations for the starch metabolism related genes in this study

Gene name	Group	Enzyme	Locus
PGI1 (PGI)		Phosphoglucoisomerase	At4g24620
PGM1 (PGM)		Phosphoglucomutase	At5g51820
APS1 (ADG1)		AGPase small subunit 1	At5g48300
APS2		AGPase small subunit-like 2	At1g05610
APL1 (ADG2)		AGPase large subunit 1	At5g19220
APL2		AGPase large subunit 2	At1g27680
APL3		AGPase large subunit 3	At4g39210
APL4		AGPase large subunit 4	At2g21590
GBSS1		Granule-bound starch synthase 1	At1g32900
SS1	Starch Synthesis	Soluble starch synthase 1	At5g24300
SS2		Soluble starch synthase 2	At3g01180
SS3		Soluble starch synthase 3	At1g11720
SS4		Soluble starch synthase 4	At4g18240
SS5		Soluble starch synthase 5	AT5G65685
MFP1		MAR-binding filament-like protein 1	AT3G16000
MRC		myosin-resembling chloroplast protein	AT4G32190
PTST		Protein targeting to starch	AT5G39790
PTST3		Protein targeting to starch 3	AT5G03420
PTST2		Protein targeting to starch 2	AT1G27070
BE1		Starch branching enzyme 1	At3g20440
BE2		Starch branching enzyme 2	At5g03650
BE3		Starch branching enzyme 3	At2g36390
GWD1 (SEX1)		Glucan, water dikinase 1	At1g10760
LDA (PU1)		Limit dextrinase	At5g04360
ISA1		Isoamylase 1	At2g39930
ISA2		Isoamylase 2	At1g03310
GWD2		Glucan, water dikinase 2	At4g24450
PWD (GWD3)		Phosphoglucan, water dikinase	At5g26570
SEX4		Starch excess 4	At3g52180
LSF2		Like Sex Four 2	At3g10940
LSF1		Like Sex Four 1	At3g01510
BAM1	Starch Breakdown	beta-Amylase 1	At3g23920
BAM2		beta-Amylase 2	At4g00490
BAM3		beta-Amylase 3	At4g17090
BAM4		beta-Amylase 4	At5g55700
BAM5		beta-Amylase 5	At4g15210
BAM6		beta-Amylase 6	At2g32290
BAM7		beta-Amylase 7	At2g45880
BAM8		beta-Amylase 8	At5g45300
BAM9		beta-Amylase 9	At5g18670
AMY1		alpha-Amylase 1	At4g25000

AMY2		alpha-Amylase 2	At1g76130
AMY3		alpha-Amylase 3	At1g69830
ISA3		Isoamylase 3	At4g09020
PHS1		Glucan phosphorylase1	At3g29320
PHS2		Glucan phosphorylase2	At3g46970
DPE1		Disproportionating enzyme1	At5g64860
DPE2		Disproportionating enzyme2	At2g40840
PGIC		Phosphoglucoisomerase	At5g42740
PGM2		Phosphoglucomutase2	At1g70730
PGM3		Phosphoglucomutase3	At1g23190
PGML1		Phosphoglucomutase-like1	At1g70820
PGML2		Phosphoglucomutase-like2	At4g11570
PGML3		Phosphoglucomutase-like3	At5g17530
AtGPT1		6-phosphate/phosphate translocator1	At5g54800
AtGPT2		6-phosphate/phosphate translocator2	At1g61800
AtGPT2-like		glucose 6 phosphate/phosphate translocator-like protein	At5g17630
TPT (TPT1)	Sugar transport	phosphate/triose-phosphate translocator precursor	At5g46110
PPT1		phosphate/phosphoenolpyruvate translocator precursor	At5g33320
PPT2		putative phosphate/phosphoenolpyruvate translocator	At3g01550

Table S2 Gene sets enriched in dY compared to dP. 9 gene sets are significant at FDR < 25%, with 8 sets up-regulated and 1 set down-regulated in dY.

Up-regulated gene sets in dY

NAME	SIZE	ES	NES	NOM p	FDR
TROPANE PIPERIDINE AND PYRIDINE ALKALOID BIOSYNTHESIS(ATH00960)	34	-0.413	-1.40003	0	0.162197
STEROID BIOSYNTHESIS(ATH00100)	35	-0.39153	-1.40959	0	0.175942
GALACTOSE METABOLISM(ATH00052)	57	-0.49128	-1.46078	0	0.176432
ARACHIDONIC ACID METABOLISM(ATH00590)	16	-0.58163	-1.3707	0	0.179975
CYANOAMINO ACID METABOLISM(ATH00460)	65	-0.4611	-1.37909	0	0.190469
ETHER LIPID METABOLISM(ATH00565)	26	-0.57869	-1.34688	0	0.199318
MAPK SIGNALING PATHWAY PLANT(ATH04016)	132	-0.52479	-1.34712	0	0.217732
BASAL TRANSCRIPTION FACTORS(ATH03022)	55	-0.54215	-1.3479	0	0.241408

Down-regulated gene sets in dY

NAME	SIZE	ES	NES	NOM p	FDR
SULFUR METABOLISM(ATH00920)	42	0.553634	1.578908	0	0.052

Table S3 Gene sets enriched in dY compared to dR. No gene set is significant at FDR < 25%. Pathways marked in red color are also identified when dY is compared to dP.

Up-regulated gene sets in dY

NAME	SIZE	ES	NES	NOM p	FDR
FLAVONOID BIOSYNTHESIS(ATH00941)	21	0.652412	1.471937	0	0.510483
SPHINGOLIPID METABOLISM(ATH00600)	27	0.674554	1.44978	0	0.368014
HOMOLOGOUS RECOMBINATION(ATH03440)	65	0.623518	1.314947	0	0.73747
NUCLEOTIDE EXCISION REPAIR(ATH03420)	69	0.518342	1.303449	0	0.648946
UBIQUITIN MEDIATED PROTEOLYSIS(ATH04120)	153	0.548055	1.282205	0	0.663912
INOSITOL PHOSPHATE METABOLISM(ATH00562)	74	0.482839	1.266197	0	0.674823
PLANT HORMONE SIGNAL TRANSDUCTION(ATH04075)	273	0.328057	1.265199	0	0.60247
AUTOPHAGY OTHER(ATH04136)	40	0.654511	1.242616	0	0.582728
**BASAL TRANSCRIPTION FACTORS(ATH03022)**	55	0.636561	1.213176	0	0.503784
ENDOCYTOSIS(ATH04144)	145	0.432642	1.209341	0	0.488744
VALINE LEUCINE AND ISOLEUCINE DEGRADATION(ATH00280)	49	0.358606	1.195963	0	0.506403
**MAPK SIGNALING PATHWAY PLANT(ATH04016)**	132	0.349692	1.167332	0	0.506405

Down-regulated gene sets in dY

NAME	SIZE	ES	NES	NOM p	FDR
NITROGEN METABOLISM(ATH00910)	42	-0.4926	-1.41321	0	0.891131
**SULFUR METABOLISM(ATH00920)**	42	-0.51413	-1.34572	0	1
PHOTOSYNTHESIS(ATH00195)	75	-0.7052	-1.33434	0	0.78618
PENTOSE AND GLUCURONATE INTERCONVERSIONS(ATH00040)	96	-0.48573	-1.31406	0	0.721116
2 OXOCARBOXYLIC ACID METABOLISM(ATH01210)	71	-0.69275	-1.28064	0	0.553171
PORPHYRIN AND CHLOROPHYLL METABOLISM(ATH00860)	52	-0.60004	-1.272	0	0.539803
CYSTEINE AND METHIONINE METABOLISM(ATH00270)	119	-0.39915	-1.25682	0	0.49996
PHENYLALANINE TYROSINE AND TRYPTOPHAN BIOSYNTHESIS(ATH00400)	53	-0.44844	-1.22244	0	0.514477
PHAGOSOME(ATH04145)	76	-0.46903	-1.21778	0	0.496866
VALINE LEUCINE AND ISOLEUCINE BIOSYNTHESIS(ATH00290)	20	-0.83347	-1.20717	0	0.530411
GLUTATHIONE METABOLISM(ATH00480)	102	-0.30217	-1.20373	0	0.510907
PHOTOSYNTHESIS ANTENNA PROTEINS(ATH00196)	21	-0.90896	-1.20119	0	0.49728
PYRIMIDINE METABOLISM(ATH00240)	58	-0.31551	-1.1884	0	0.491552
GLUCOSINOLATE BIOSYNTHESIS(ATH00966)	24	-0.86437	-1.18308	0	0.49051

Table S4 Primers used for qRT-PCR

Name	Primer1	Primer2
BE3	ACCGTATCGGTTGCTCTGTTCC	CTATCGTCGTGCCTTCCATCAG
ISA1	TCCCTCATGGTTTCCCAAGGTG	TGGCAATACGTGTTGTTGTTTCCC
ISA3	GGAATGGGATGTACCGGGATGATG	CTTTCATGCCACTGTCACCCTTG
GBSS1	ACCAAGACTTCTCCTGGAAGGG	GCTTCACTTCCCGCCACATTAAG
GPT2	TCTATTGCCGTGGAAGGTCCTC	ACTTTGTGCCACTACCCACCAG
PGM1	ATTTCCGTGCTGGTCCTAAGGG	TTCTCGGCAACACGATCAAGAGC
SS4	GAGTCAAGACGGCCTTTGGTTG	TATGTGGAACCGGGCTAGAACC
DPE1	ACGCGCACAGGACTTGTATGAC	ACCATGGCAACTTTCGCTTCAG
